# Dose Dependence Effect in Biallelic *WNT10A* Variant-Associated Tooth Agenesis Phenotype

**DOI:** 10.3390/diagnostics12123087

**Published:** 2022-12-07

**Authors:** Haochen Liu, Bichen Lin, Hangbo Liu, Lanxin Su, Hailan Feng, Yang Liu, Miao Yu, Dong Han

**Affiliations:** 1 Department of Prosthodontics, Peking University School and Hospital of Stomatology & National Center of Stomatology & National Clinical Research Center for Oral Diseases & National Engineering Research Center of Oral Biomaterials and Digital Medical Devices, Beijing 100081, China; 2 First Clinical Division, Peking University School and Hospital of Stomatology & National Center of Stomatology & National Clinical Research Center for Oral Diseases & National Engineering Research Center of Oral Biomaterials and Digital Medical Devices, Beijing 100081, China

**Keywords:** *WNT10A*, biallelic variants, odonto-onycho-dermal dysplasia syndrome (OODD), nonsyndromic tooth agenesis

## Abstract

The goal of this study was to identify the pathogenic gene variants in patients with odonto-onycho-dermal dysplasia syndrome (OODD) or nonsyndromic tooth agenesis. Four unrelated individuals with tooth agenesis and their available family members were recruited. Peripheral blood was collected from four probands and five family members. Whole-exome sequencing (WES) and Sanger sequencing were used to identify the pathogenic gene variants. The harmfulness of these variations was predicted by bioinformatics. We identified four biallelic variants of the *WNT10A* gene in four patients, respectively: the proband#660: c.1176C > A (p.Cys392*) and c.812G > A (p.Cys271Tyr); the proband#681: c.637G > A (p.Gly213Ser) and c.985C > T (p.Arg329*); the proband#829: c.511C > T (p.Arg171Cys) and c.637G > A (p.Gly213Ser); and the proband#338: c.926A> G (p.Gln309Arg) and c.511C > T (p.Arg171Cys). Among them, two variants (c.812G > A; p.Cys271Tyr and c.985C > T; p.Arg329*) were previously unreported. Bioinformatics analysis showed that the pathogenicity of these six variants was different. Tertiary structure analysis showed that these variants were predicted to cause structural damage to the WNT10A protein. Genotype–phenotype analysis showed that the biallelic variants with more harmful effects, such as nonsense variants, caused OODD syndrome (#660 Ⅱ-1) or severe nonsyndromic tooth agenesis (NSTA) (#681 Ⅱ-1); the biallelic variants with less harmful effects, such as missense variants, caused a mild form of NSTA (#829 Ⅱ-2 and #338 Ⅱ-1). Individuals with a heterozygous variant presented a mild form of NSTA or a normal state. Our results further suggest the existence of the dose dependence of *WNT10A* pathogenicity on the tooth agenesis pattern, which broadens the variation spectrum and phenotype spectrum of *WNT10A* and could help with clinical diagnosis, treatment, and genetic counseling.

## 1. Introduction

Tooth agenesis (TA), also known as congenitally missing teeth, is one of the most common developmental anomalies in the dental clinic [1]. Many studies have reported that the prevalence of TA varies from 2% to 10% (excluding the third molar) among different populations [2,3,4]. The prevalence of TA was found to be 5.89% in the Chinese population [5]. TA can lead to malocclusion, masticatory dysfunction, speech changes, and aesthetic problems [6]. TA can either occur as an isolated condition (nonsyndromic TA/NSTA) or can be associated with a syndrome (syndromic TA), highlighting the heterogeneity of the condition [7].

The human wingless-type *MMTV* integration site family member 10A (*WNT10A*; OMIM *606268) is an important member of the *WNT* gene family that mediates the transcriptional activation of the canonical WNT signaling pathway (WNT/β-catenin) [8,9]. It is well established that variants in the *WNT10A* gene can cause syndromic TA (odonto-onycho-dermal dysplasia syndrome (OODD; OMIM#257980) and Schöpf–Schulz–Passarge syndrome (SSPS; OMIM#224750)), and nonsyndromic TA (STHAG4; OMIM#150400) [10,11,12,13,14,15,16,17].

OODD is an autosomal recessive multiple organ disorder characterized by severe tooth agenesis, dry hair, smooth tongue, smooth tongue with marked reduction in fungiform and filiform papillae, onychodysplasia, hyperkeratosis of the palms and soles, hypo- and hyperhidrosis of the skin, and patches on the face [10,15,18,19]. In 2007, Adaimy et al. first identified a homozygous nonsense variant, c.697G > T; p.E233X, in the *WNT10A* gene in a family with autosomal recessive OODD, confirming that the *WNT10A* gene is the causative gene of OODD [10]. SSPS is an autosomal recessive disorder characterized by a constellation of multiple eyelid cysts, hypodontia, hypotrichosis, palmoplantar hyperkeratosis, and onychodystrophy [20]. In 2009, Bohring et al. first reported that a *WNT10A* variant can lead to SSPS [11,21]. In 2011, *WNT10A* was identified as the pathogenic gene of nonsyndromic tooth agenesis (NSTA) [22]. Van Den et al. reported that *WNT10A* variants were identified in 56% of cases with NSTA [23]. The results of our previous study also showed that *WNT10A* variants were detected in 15.8% of NSTA patients with 1–3 missing teeth and 51.6% of NSTA patients with four or more missing teeth [12].

The aim of this study was to identify the pathogenic gene variants in patients with OODD or NSTA, and analyze the genotype–phenotype distinction.

## 2. Methods

### 2.1. Study Subjects

Four unrelated individuals with tooth agenesis and their available family members (5 in total) were recruited from the Department of Prosthodontics at the Peking University School and Hospital of Stomatology. The patients and the available family members were examined for possible clinical symptoms in hair, skin, nails, and the intra-oral region. Panoramic radiographs were used to determine the number of instances of tooth agenesis. Written informed consent from all the participants for the use of blood and clinical data, and publication of their photographs, was obtained. This study was approved by the Ethics Committee of the Peking University School and Hospital of Stomatology (PKUSSIRB-202162021).

### 2.2. Variant Detection

The BioTek DNA Whole-Blood Mini Kit (BioTek, Beijing, China) was used to extract genomic DNA from peripheral blood, according to the manufacturer’s instructions. Whole-exome sequencing (WES) was performed in the four probands and five available family members by Beijing Angen Gene Medicine Technology (Beijing, China) by using the Illumina-X10 platform to identify potential pathogenic gene variants. To filter the detected variants, orodental-related genes were annotated [24]. Then, we screened the potential variants according to the methods described in previous studies [25,26]. Briefly, based on the WES results, we filtered all nonsynonymous single-nucleotide variants and insertions/deletions according to the MAF ≤ 0.01 in the databases, including the single nucleotide polymorphism database (dbSNP, http://www.ncbi.nlm.nih.gov/projects/SNP/snp_summary.cgi, accessed on 7 September 2022), the 1000 Genomes Project data in Ensembl (http://asia.ensembl.org/Homo_sapiens/Info/Index, accessed on 7 September 2022), the Genome Aggregation Database (gnomAD, http://gnomad.broadinstitute.org, accessed on 7 September 2022) and the Exome Aggregation Consortium (ExAC, http://exac.broadinstitute.org, accessed on 7 September 2022). The candidate genes and variants left after filtering showed in Supplementary Table S1. Then the variants that affect protein function were predicted based on the results obtained from Mutation Taster (http://www.mutationtaster.org, accessed on 7 September 2022), Functional Analysis through Hidden Markov Models v2.3 (Fathmm, http://fathmm.biocompute.org.uk, accessed on 7 September 2022), and Poly-morphism Phenotyping v2 (PolyPhen-2, http://genetics.bwh.harvard.edu/pph2/, accessed on 7 September 2022).

### 2.3. Sanger Sequencing and Clone Sequencing

To verify the WES results, Sanger sequencing was used to sequence the related *WNT10A* (NM_025216.2) fragments. The exons of the *WNT10A* gene and the intron–exon boundaries were amplified by polymerase chain reaction (PCR) with specific primers (primers provided in Supplementary Table S2). The PCR products were sequenced by Tsingke Biological Technology (Beijing, China) and further cloned into the pClone007 Simple Vector (Tsingke, Beijing, China) to identify the exact status of the variant.

### 2.4. Bioinformatics Analysis

For conservation analysis, the amino acid sequences of WNT10A (NP_079492.2) among different species were obtained from the NCBI database (https://www.ncbi.nlm.nih.gov/, accessed on 7 September 2022). Molecular Evolutionary Genetics Analysis Version 11 (MEGA 11.0) [27] was used to conduct the multiple sequence alignment, and the sequence logos were performed with WebLogo V2.8.2 (http://weblogo.berkeley.edu/, accessed on 7 September 2022).

The protein structure of WNT10A was predicted by using the AlphaFold Protein Structure Database (https://alphafold.ebi.ac.uk/, accessed on 7 September 2022). PyMol v2.1 (Molecular Graphics System, DeLano Scientific, San Carlos, CA, USA) was used to analyze the three-dimensional structural changes caused by the variants.

## 3. Results

### 3.1. Clinical Findings

**Figure 1:**
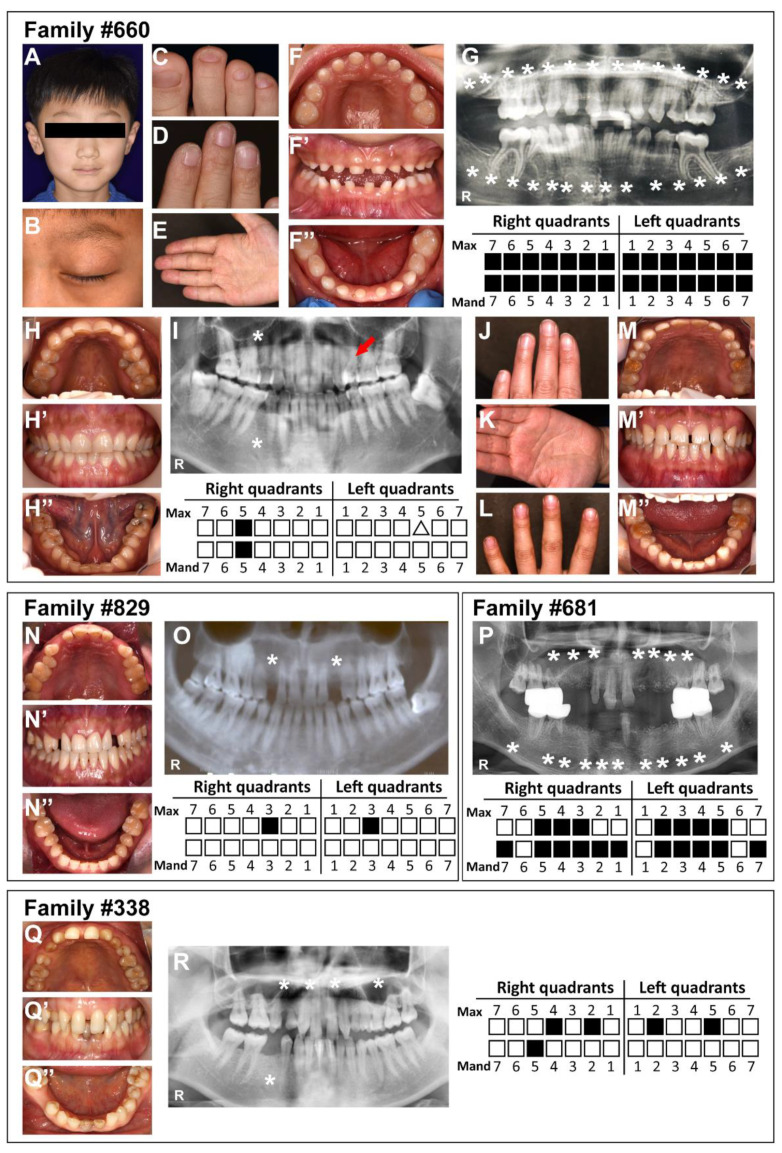
Clinical photographs and panoramic radiographs of four families. (**A**–**E**) Photographs of the face, feet, and hand of proband #660. (**F**–**G**) Intra-oral photographs, panoramic radiograph, and schematic of proband #660. (**H**,**I**) Intra-oral photographs, panoramic radiograph, and schematic of mother of proband #660. (**J**,**K**) Photographs of the hand of the mother of proband #660. (**L**) Photographs of the hand of the father of the proband of family #660. (**M**–**M’’**) Intra-oral photographs of the father of proband #660. (**N**,**O**) Intra-oral photographs, panoramic radiograph, and schematic of proband #829. (**P**) Panoramic radiograph and schematic of proband #681. (**Q**,**R**) Intra-oral photographs, panoramic radiograph, and schematic of proband #338. Asterisks and solid squares indicate congenitally missing teeth; the red arrow and triangle indicate malformed tooth; Max: maxillary; Mand: mandibular.

The proband of family#660 was a 9-year-old boy who presented agenesis of all permanent teeth, hyperkeratotic palms and soles and dystrophic toenails and fingernails, and sparse hair (Figure 1A–G). His mother was affected by NSTA (Figure 1H–K). His father did not have any obvious phenotypic abnormalities (Figure 1L–M”).

The proband of family#829 was a 33-year-old male who presented with NSTA (Figure 1N–O). He showed two permanent teeth missing, without any symptoms of ectodermal dysplasia. According to the patient’s description, he had a sister with NSTA and his parents were normal.

The proband of family#681 was an 18-year-old male who presented with NSTA (Figure 1P). He was congenitally missing 22 permanent teeth. His skin, hair, eyebrows, and eyelashes were found to be developed normally, and no other ectodermal organs, such as sweat glands or sebaceous glands, were found to be maldeveloped. His parents and younger brother did not show any tooth agenesis or other abnormalities of ectodermal hypoplasia.

The proband of family#338 was a 31-year-old female with NSTA (Figure 1Q–R). She showed five permanent teeth missing and no symptoms of ectodermal dysplasia. According to her description, her parents had no abnormalities.

### 3.2. The Identification of WNT10A Variants

**Figure 2:**
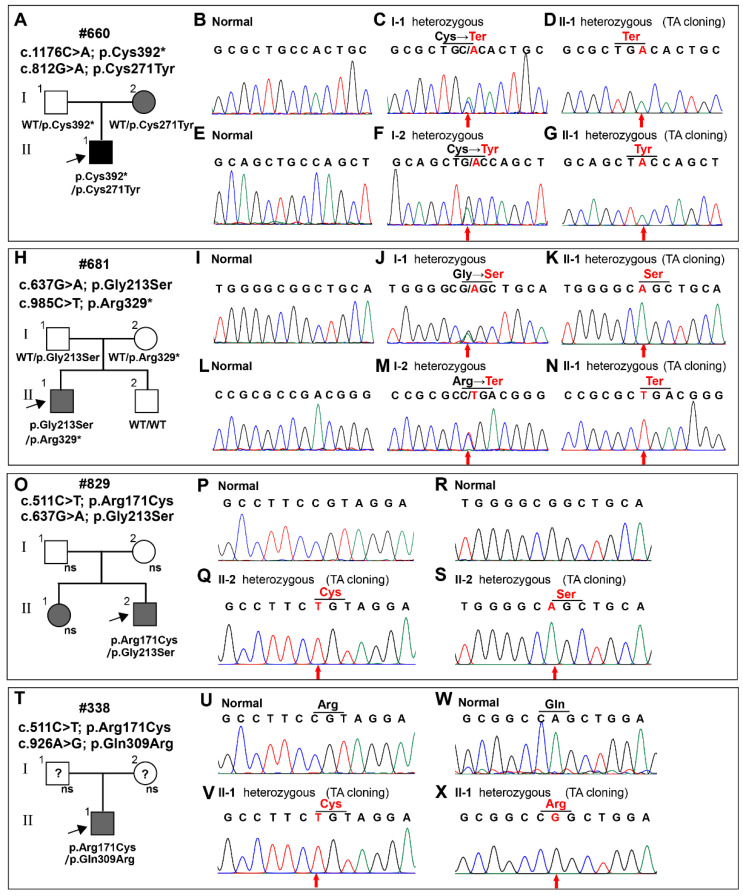
Family pedigrees and sequencing chromatograms of the four families in this study. (**A**–**G**) Sequencing chromatograms of available DNA in family #660. The proband carried one nonsense variant (*WNT10A*: c.1176C > A; p.Cys392*), which was inherited from his father (Ⅰ-1), and one missense variant (*WNT10A*: c.812G > A; p.Cys271Tyr), which was inherited from his mother (Ⅰ-2). (**H**–**N**) Sequencing chromatograms of available DNA in family #681. The proband carried one missense variant (*WNT10A*: c.637G > A; p.Gly213Ser), which was inherited from his father (Ⅰ-1), and one nonsense variant (*WNT10A*: c.985C > T; p.Arg329*), which was inherited from his mother (Ⅰ-2). (**O**–**S**) Sequencing chromatograms of available DNA in family #829. The proband carried two missense variants (*WNT10A*: c.511C > T; p.Arg171Cys and c.637G > A; p.Gly213Ser) on different alleles. (**T**–**X**) Sequencing chromatograms of available DNA in family #338. The proband carried two missense variants (*WNT10A*: c.511C > T; p.Arg171Cys and c.926A > G; p.Gln309Arg) on different alleles. Solid circles and squares represent the individuals with OODD. Grey circles and squares represent the individuals with NSTA. Black arrows indicate the probands. Red arrows indicate the variants. NS indicates that DNA samples are unavailable. Question marks indicate that the phenotype is untraceable.

We identified four pair biallelic variants of the *WNT10A* gene in these patients by WES and Sanger sequencing. An overview of these variants, with amino acid changes and possible impacts, is presented in Table 1. The proband of family #660 carried one nonsense variant (c.1176C > A; p.Cys392*) inherited from his father, and one missense variant (c.812G > A; p.Cys271Tyr), inherited from his mother (Figure 2A–G). The phenotype of this family is consistent with the autosomal recessive genetic model of OODD.

The proband of family #681 carried one missense variant (c.637G > A; p.Gly213Ser) inherited from his father, and one nonsense variant (985C > T; p.Arg329*) inherited from his mother (Figure 2H–N). However, his brother did not carry either of the two variants.

In family #829, the proband carried two missense variants (c.511C > T; p.Arg171Cys and c.637G > A; p.Gly213Ser) on different alleles (Figure 2O–S). Unfortunately, we did not obtain DNA samples from his parents or sister.

The proband #338 also had two missense variants (c.511C > T; p.Arg171Cys and c.926A > G; p.Gln309Arg) on different alleles (Figure 2T–X), but the sources of the variants could not be determined, because DNA samples from his parents were unavailable. Details of the genotypes and phenotypes of these four families were shown in Table 2.

### 3.3. Bioinformatics Analysis of the WNT10A Variants

According to Mutation Taster, Fathmm, PolyPhen-2, gnomAD, dbSNP, 1000G, and the classification of pathogenic variants with the standards of the 2015 American College of Medical Genetics and Genomics and the Association for Molecular Pathology (ACMG), c.1176C > A (p.Cys392*) and c.985C > T(p.Arg329*) were predicted to be pathogenic, whereas c.812G > A(p.Cys271Ty) was predicted to be likely pathogenic. However, the pathogenicity of c.511C > T(p.Arg171Cys), c.637G > A(p.Gly213Ser) and c.926A > G(p.Gln309Arg) was of uncertain significance in the current data and requires further investigation (Table 1).

**Figure 3:**
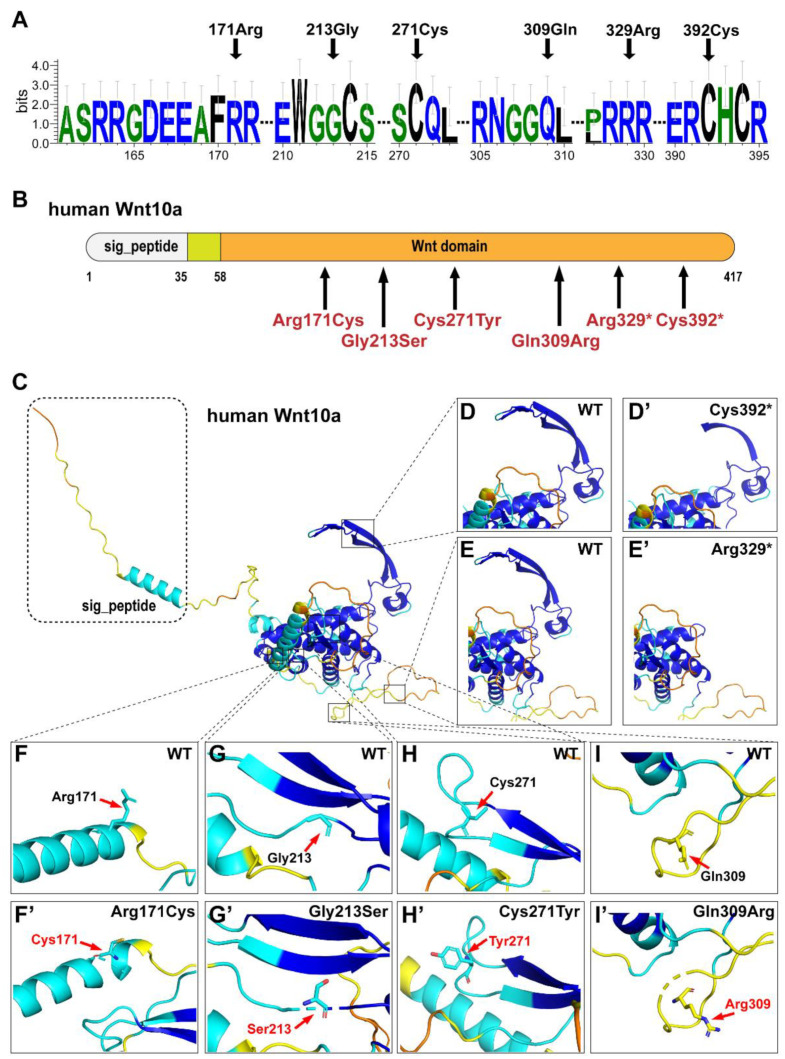
Bioinformatics analysis of the WNT10A variants. (**A**) Conservation analysis of the WNT10A amino acid sequences among different species. (**B**) Schematic diagram of the wild type WNT10A protein and the localization of the six WNT10A variants identified in this study. (**C**) Structure of wild-type WNT10A. (**D**,**D’**) Structure of wild type and Cys392* in WNT10A. (**E**,**E’**) Structure of wild type and Arg392* in WNT10A. (**F**,**F’**) Structure of Arg171 and Cys171 in WNT10A. (**G**,**G’**) Structure of Gly213 and Ser213 in WNT10A. (**H**,**H’**) Structure of Cys271 and Tyr271 in WNT10A. (**I**,**I’**) Structure of Gln309 and Arg309 in WNT10A.

Based on the results of conservation analyses in multiple species, 171Arg, 213Gly, 217Cys, 309Gln, 329Arg, and 392Cys were highly conserved (Figure 3A). Six variants (p.Arg171Cys, p.Gly213Ser, p.Cys271Tyr, p.Gln309Arg, p.Arg329*, and p.C392*) were located in the highly conserved Wnt domain of WNT10A (Figure 3B).

The Cys392* resulted in a truncated protein losing 25 amino acid residues from the C-terminus (Figure 3D,D’). The Arg329* resulted in a truncated protein losing 88 amino acid residues from the C-terminus (Figure 3E,E’). The variant Arg171Cys resulted in a basic residue arginine, which had a positively charged side chain, being substituted with cysteine, which had a side chain capable of forming a disulfide bond with another cysteine (Figure 3F,F’). The variant Gly213Ser resulted in a basic residue glycine, which had no side chain, being substituted with serine, which had a neutral side chain (Figure 3G,G’). For the variant Cys271Tyr, the residue at sequence position 271 in this protein is a cysteine, which has a side chain capable of forming a disulfide bond with another cysteine, and hence provides strong structural support for the protein; the variant residue is a tyrosine with an aromatic side chain, which can stack against others (Figure 3H,H’). For the variant Gln309Arg, the residue at sequence position 309 in this protein is a glutamine with a neutral side chain; the variant residue is an arginine, which has a positively charged side chain, making it hydrophilic (Figure 3I,I’).

## 4. Discussion

TA is one of the most common developmental diseases in oral clinics. In clinical treatment, we usually need to develop a multidisciplinary treatment plan, especially orthodontic, prosthetic, and implant replacement therapy for the rehabilitation of different areas of the dental arch. In addition, the identification of genetic factors may be particularly useful for early prediction of this condition and for the development of prevention strategies and novel treatments in the future.

Human tooth development is a long and complex process which involves a series of reciprocal and sequential interactions between the embryonic stomodeal epithelium and the underlying neural crest-derived mesenchyme [28]. Despite environmental and epigenetic factors, tooth development is mainly controlled by genes. So far, more than 200 genes have been found to be involved in tooth development. Tooth development is a dynamic process, including bud stage, cap stage, bell stage, root development and tooth eruption [29]. The Wnt/β-catenin signaling pathway is involved in the development of multiple organs and is temporally and spatially activated in the tooth formation zone at all stages of tooth development, suggesting its important role in tooth formation [30]. The mechanisms of the Wnt pathway include extracellularly secreted glycoproteins (19 human-level Wnt ligands), seven transmembrane spanning receptors (Frizzled and LRP5/6), cytoplasmic proteins (DVL, APC, AXIN, GSK3 β and β-linked proteins), nuclear transcription factors (TCF/LEF) and several Wnt-related molecules (MSX1, DKK1, KREMEN1 and ANTXR1) [8]. The genetic link between the TA and Wnt pathways was first confirmed by identifying variants in the axis inhibitor 2 (*AXIN2*) gene in a family with syndromic TA [31]. *AXIN2*, an intracellular inhibitor of Wnt/β-catenin signaling, is highly expressed in enamel knots and mesenchymal dental cells during tooth formation. A large number of variants in genes encoding Wnt ligands (e.g., *WNT10A* and *WNT10B*) and associated receptors (*LRP6* and *KREMEN1*) were recently discovered by whole exome and Sange sequencing in patients with TA. Among them, *WNT10A* was the second-most frequently variant gene in individuals with NSTA [8].

In this study, we reported an OODD cases (#660 Ⅱ-1) and three NSTA cases (#829 Ⅱ-2, #681Ⅱ-1, and #338 Ⅱ-1) caused by biallelic *WNT10A* variants. In total, six different *WNT10A* variants were identified, including two novel variants (c.812G > A; p.Cys271Tyr and c.985C > T; p.Arg329*) and four reported variants (c.1176C > A; p.Cys392*, c.511C > T; p.Arg171Cys, c.637G > A; p.Gly213Ser and c.926A > G; p.Gln309Arg) [12,15,32]. These patients carried biallelic *WNT10A* variants; however, the phenotypes are extremely different.

The proband (#660 Ⅱ-1) with a biallelic pathogenic *WNT10A* variant (c.1176C > A; p.Cys392* and c.812G > A; p.Cys271Tyr) exhibited severe syndromic tooth agenesis with classical OODD features, characterized by agenesis of all permanent teeth, hyperkeratotic palms and soles, dystrophic toenails and fingernails, and sparse hair. His two variants were inherited from his father and mother, respectively. His mother carried the heterozygous nonsense variant (c.1176C > A), which led to a more significant structural impact, showing NSTA (missing two permanent teeth), whereas his father, who carried the heterozygous missense variant (c.812G > A), was normal. The three genotypes of this family resulted in three significantly different phenotypes, suggesting that the pathogenicity of *WNT10A* may be dose dependent.

Interestingly, the other three probands (#829 Ⅱ-2, #681Ⅱ-1, and #338 Ⅱ-1) with biallelic *WNT10A* variants were diagnosed with NSTA. The proband #829 carried two missense variants (c.511C > T; p.Arg171Cys and c.637G > A; p.Gly213Ser). The proband #681 carried one nonsense variant (c.985C > T; p.Arg329*) and one missense variant (c.637G > A; p.Gly213Ser). The proband #338 carried two missense variants (c.926A > G; p.Gln309Arg and c.511C > T; p.Arg171Cys). It could be found that these three patients carried at least either the c.511C > T or c.637G > A variant. These two variants were highly frequent in Asian populations. According to multiple genetic databases, Kanchanasevee1 et al. observed the allele frequencies of *WNT10A* c.511C > T and c.637G > A variants in Asian populations up to 0.033 and 0.029, respectively, compared with those in non-Asians, which are 0.000–0.001 [33]. Our previous study showed that c.511C > T and c.637G > A were present in both tooth agenesis cases and normal controls, suggesting that these two variants might not necessarily lead to tooth agenesis, but might be risk factors for tooth agenesis [12]. A previous study based on a Japanese population showed that the population frequency of the c.637G > A variant is concentrated within tooth agenesis patients (16.0%; 8/50) rather than the general Japanese population (3.0%; 70/2318) [34]. Moreover, it was suggested that the heterozygous c.511C > T or c.637G > A allele could be a contributing factor for NSTA with low penetrance, while biallelic variants are associated with greater clinical severity [33]. In line with our predecessors’ conclusions, our results further confirmed that c.511C > T and c.637G > A were risk factors for tooth agenesis, and they aggravate the phenotype of NSTA when combined with other variants.

According to bioinformatics analysis and ACMG classification, c.1176C > A (p.Cys392*) and c.985C > T(p.Arg329*) were predicted to be pathogenic; c.812G > A(p.Cys271Ty) was predicted to be likely pathogenic; c.511C > T(p.Arg171Cys), c.637G > A(p.Gly213Ser) and c.926A > G(p.Gln309Arg) was predicted to be uncertain significance. The severity of the clinical phenotype in this study was consistent with the ACMG classification. This suggests that bioinformatics analysis can be used as a method to predict the pathogenicity of variants in *WNT10A*.

Many TA-associated pathogenic genes were phenotypically dose dependent, leading to syndromic TA when pathogenicity was strong and NSTA when pathogenicity was weak [35]. The phenotypic spectrum of *WNT10A* variants suggested a dosage-dependent pattern with variable expression in a Korean family [36]. In this study, the biallelic variants with more harmful effects, such as nonsense variants, caused OODD syndrome (#660 Ⅱ-1) or severe NAST (#681 Ⅱ-1); the biallelic variants with less harmful effects, such as missense variants, caused a mild form of NSTA (#829 Ⅱ-2 and #338 Ⅱ-1); individuals with a heterozygous variant presented NSTA or a normal state. Our results further confirmed the existence of the dose dependence of *WNT10A* pathogenicity on the tooth agenesis pattern. However, there were some limitations in our study due to the sample size. If more samples of biallelic *WNT10A* variant were available, we would be able to precisely determine the phenotype. In addition, performing functional experiments could also help us to better determine the degree of pathogenicity of the variant. All these need to be improved in our future studies.

## 5. Conclusions

In conclusion, we reported four rare biallelic *WTN10A* variants in patients with OODD or NSTA. Our results broaden the variation spectrum and phenotype spectrum of *WNT10A*, which could help with clinical diagnosis, treatment, and genetic counseling. However, the pathogenic mechanism of *WNT10A*-associated tooth agenesis is still unclear and needs to be further studied.

## Figures and Tables

**Table 1:** Pathogenic prediction of the *WNT10A* variants identified in this study.

Variant	Proband	Domain	Mutation Taster	Fathmm	PolyPhen-2	gnomAD, dbSNP, 1000G	ACMG Classification (Evidence of Pathogenicity)
c.1176C > A/ p.Cys392*	#660 II-1	Wnt	Disease causing	-	-	rs1553623389	Pathogenic PVS1 + PP1 + PP5
c.812G > A/ p.Cys271Tyr	#660 II-1	Wnt	Disease causing	DAMAGING (−2.18)	Probably damaging (1.000)	Not found	Likely pathogenic PM2 + PM3 + PP1 + PP2 + PP3
c.511C > T/ p.Arg171Cys	#829 II-2 #338 II-1	Wnt	Disease causing	TOLERATED (−1.04)	Probably damaging (0.999)	rs116998555	Uncertain significance BP6
c.637G > A/ p.Gly213Ser	#829 II-2 #681 II-1	Wnt	Disease causing	DAMAGING (−1.66)	Probably damaging (1.000)	rs147680216	Uncertain significance PP1 + PP3
c.985C > T/ p.Arg329*	#681 II-1	Wnt	Disease causing	-	-	Not found	Pathogenic PVS1 + PM2 + PP1 + PP2
c.926A > G/ p.Gln309Arg	#338 II-1	Wnt	Disease causing	TOLERATED (−0.92)	Benign (0.006)	rs1461989045	Uncertain significance PP3 + PM1 + BS4 + BP2

PVS1: Null variant (nonsense, frameshift, canonical ±1 or 2 splice sites, initiation codon, single or multiexon deletion) in a gene where LOF is a known mechanism of disease. PP1: Cosegregation with disease in multiple affected family members in a gene definitively known to cause the disease. PP5: Reputable source recently reports variant as pathogenic, but the evidence is not available to the laboratory to perform an independent evaluation. PM2: Absent from controls (or at extremely low frequency if recessive) in Exome Sequencing Project, 1000 Genomes Project, or Exome Aggregation Consortium. PM3: For recessive disorders, detected in *trans* with a pathogenic variant. PP2: Missense variant in a gene that has a low rate of benign missense variation and in which missense variants are a common mechanism of disease. PP3: Multiple lines of computational evidence support a deleterious effect on the gene or gene product. BP6: Reputable source recently reports variant as benign, but the evidence is not available to the laboratory to perform an independent evaluation. PM1: Located in a mutational hot spot and/or critical and well-established functional domain (e.g., active site of an enzyme) without benign variation. BS4: Lack of segregation in affected members of a family. BP2: Observed in trans with a pathogenic variant for a fully penetrant dominant gene/disorder or observed in cis with a pathogenic variant in any inheritance pattern.

**Table 2:** Details of the genotypes and phenotypes of the four families in this study.

Origin	Variation	Variation Type	Disease		Right Quadrants								Left Quadrants								Permanent Tooth Missing Number
				Max		7	6	5	4	3	2	1	1	2	3	4	5	6	7		
				Mand		7	6	5	4	3	2	1	1	2	3	4	5	6	7		
#660 II-1	p.Cys392* p.Cys271Tyr	Biallelic	OODD			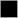	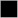	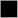	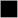	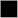	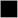	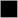	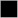	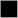	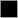	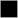	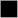	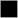	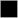		28
						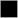	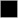	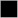	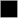	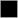	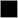	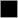	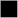	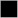	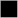	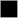	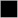	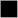	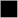		
#660 I-1	WT p.Cys392*	Monoallelic	Normal			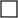	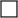	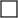	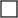	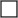	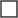	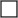	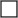	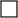	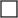	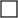	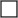	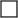	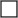		0
						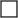	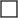	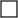	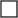	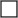	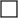	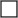	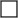	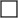	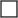	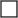	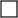	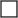	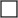		
#660 I-2	WT p.Cys271Tyr	Monoallelic	NSTA			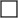	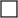	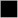	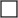	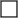	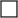	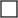	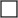	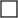	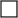	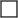	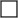	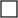	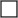		2
						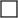	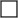	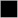	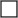	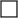	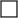	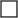	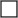	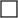	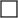	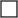	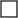	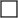	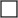		
#829 II-2	p.Arg171Cys p.Gly213Ser	Biallelic	NSTA			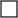	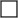	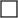	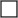	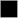	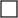	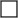	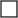	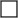	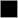	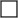	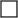	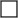	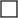		2
						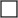	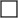	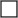	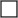	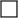	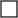	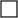	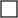	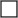	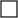	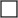	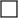	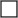	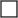		
#829 II-1	NA	NA	NSTA			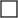	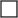	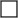	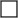	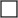	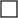	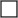	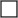	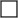	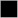	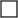	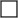	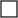	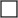		1
						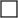	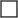	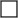	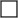	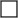	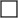	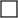	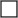	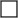	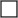	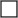	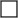	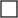	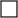		
#829 I-1	NA	NA	Normal			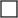	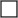	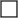	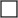	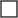	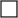	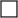	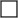	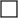	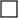	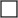	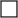	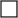	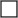		0
						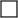	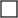	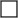	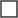	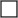	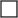	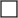	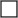	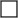	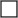	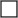	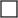	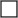	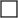		
#829 I-2	NA	NA	Normal			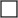	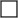	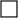	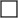	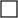	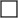	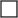	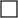	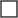	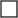	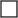	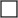	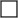	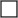		0
						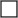	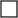	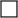	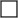	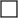	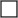	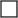	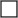	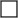	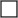	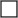	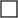	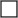	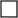		
#681 II-1	p.Gly213Ser p.Arg329*	Biallelic	NSTA			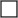	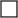	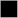	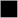	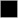	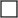	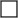	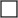	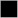	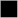	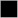	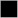	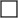	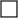		18
						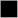	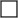	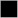	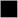	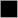	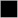	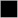	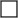	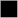	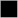	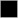	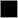	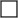	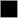		
#681 II-2	WT WT	Normal	Normal			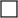	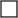	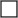	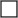	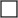	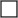	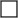	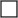	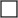	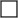	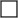	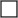	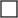	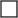		0
						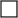	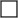	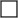	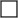	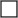	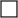	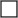	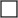	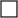	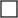	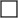	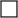	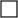	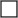		
#681 I-1	WT p.Gly213Ser	Monoallelic	Normal			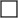	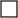	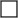	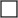	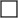	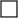	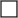	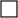	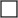	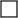	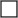	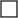	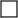	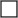		0
						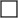	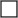	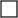	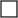	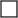	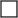	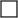	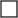	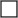	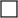	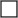	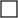	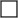	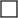		
#681 I-2	WT p.Arg329*	Monoallelic	Normal			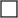	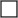	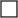	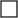	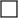	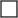	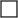	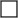	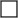	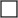	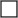	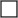	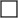	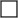		0
						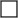	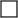	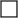	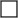	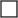	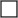	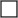	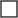	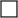	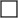	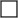	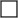	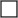	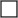		
#338 II-1	p.Arg171Cys p.Gln309Arg	Biallelic	NATA			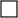	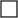	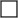	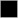	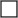	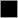	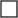	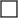	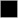	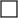	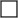	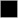	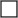	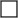		5
						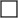	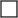	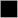	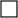	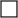	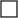	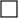	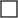	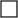	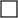	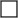	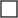	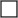	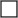		
#338 I-1	NA	NA	NA		NA																NA
#681 II-2	NA	NA	NA		NA																NA

NA, not available; solid squares, congenitally missing teeth; max, maxillary; mand, mandibular.

## Data Availability

The variations identified in this study were submitted to the ClinVar database (submission ID SCV002569146.1 and SUB11937736).

## Supplementary Materials

The following supporting information can be downloaded at: https://www.mdpi.com/article/10.3390/diagnostics12123087/s1. Table S1. The candidate genes and variants left after filtering. Table S2. The primer sequences used for PCR in this study.
