# Supplementary material for: Dose Dependence Effect in Biallelic WNT10A Variant-Associated Tooth Agenesis Phenotype
Source: Diagnostics (Basel). 2022 Dec 7;12(12):3087. doi: 10.3390/diagnostics12123087 (PMC9776737; doi:10.3390/diagnostics12123087)
Supplement: Supplementary file 1 [file diagnostics-12-03087-s001.zip › Table S1 .pdf]

Table S1 The candidate genes and variants left after filtering

Probands	Genes	chromosomal position	Variants	type
660	*SLC39A5*	chr12:56625199	NM_173596:c.141C>G (p.Tyr47X)	heterozygous
660	*ACADS*	chr12:121176179	NM_000017:c.721C>T (p.Leu241Phe)	heterozygous
660	*ADAMTS 17*	chr15:100589062	NM_139057:c.2591G>A (p.Arg864Gln)	heterozygous
660	*ADCY5*	chr3:123047501	NM_183357:c.1795G>A (p.Gly599Ser)	heterozygous
660	*ASPM*	chr1:197072955	NM_018136:c.5426A>C (p.Gln1809Pro)	heterozygous
660	*ATXN2*	chr12:111963007	NM_002973:c.1165G>A (p.Glu389Lys)	heterozygous
660	*DPYS*	chr8:105463544	NM_001385:c.353G>A (p.Arg118Gln)	heterozygous
660	*EBP*	chrX:48382441	NM_006579:c.282A>C (p.Gln94His)	hemizygous
660	*FAT4*	chr4:126336495	NM_001291303:c.6377C>T (p.Ala2126Val)	heterozygous
660	*KMT2D*	chr12:49442542	NM_003482:c.4031T>C (p.Ile1344Thr)	heterozygous
660	*LIPA*	chr10:91005469	NM_000235:c.193C>T (p.Arg65X)	heterozygous
660	*LPAR6*	chr13:48986373	NM_005767:c.187G>C (p.Asp63His)	heterozygous
660	*NSMCE2*	chr8:126194551	NM_001349487:c.471_474del (p.*158delinsEQPQLTD FIKFAYLLGLILILSCV LLLLQFCILSNFWFKS ACSFLKANSFHKVLR KFPSS*)	heterozygous
660	*PIGM*	chr1:160000604	NM_145167:c.926A>G (p.Tyr309Cys)	heterozygous
660	*PSENEN*	chr19:36237692	NM_172341:c.250C>T (p.Arg84Cys)	heterozygous
660	*RNF168*	chr3:196229742	NM_152617:c.301+2T>C (splicing)	heterozygous
660	*SHPK*	chr17:3526750	NM_013276:c.530C>T (p.Thr177Ile)	heterozygous

660	*SOX10*	chr22:38369909	NM_006941:c.994G>T (p.Ala332Ser)	heterozygous
660	*STXBP2*	chr19:7707328	NM_001272034:c.841G>T (p.Gly281Trp)	heterozygous
660	*TECTA*	chr11:121023662	NM_005422:c.4178G>A (p.Arg1393His)	heterozygous
660	*UQCRQ*	chr5:132202683	NM_014402:c.110A>G (p.Asn37Ser)	heterozygous
660	*VWF*	chr12:6127887	NM_000552:c.4697G>A (p.Arg1566Gln)	heterozygous
660	*ATM*	chr11:108216635	NM_000051:c.8584G>A (p.V2862I)	heterozygous
681	*GLDC*	chr9:6644659	NM_000170:c.289G>A (p.A97T)	heterozygous
681	*HPS5*	chr11:18313118	NM_181507:c.2311G>A (p.E771K)	heterozygous
681	*ITCH*	chr20:32981664	NM_001324197:c.47T>A (p.M16K)	heterozygous
681	*JAG1*	chr20:10622196	NM_000214:c.2828C>T (p.P943L)	heterozygous
681	*KMT2A*	chr11:118373728	NM_001197104:c.7121A>G (p.H2374R)	heterozygous
681	*MGP*	chr12:15035978	NM_001190839:c.173C>G (p.P58R)	heterozygous
681	*NPRL2*	chr3:50386910	NM_006545:c.355G>A (p.V119M)	heterozygous
681	*OTOG*	chr11:17653708	NM_001277269:c.7043C>G (p.T2348S)	heterozygous
681	*PDE3A*	chr12:20766618	NM_000921:c.1253A>T (p.K418M)	heterozygous
681	*PLEC*	chr8:144994757	NM_201380:c.9643G>A (p.V3215M)	heterozygous
681	*POLD1*	chr19:50919758	NM_001308632:c.3004G>A (p.E1002K)	heterozygous
681	*TGFBI*	chr5:135383108	NM_000358:c.770G>A (p.R257Q)	heterozygous
681	*TRIM2*	chr4:154216780	NM_001351056:c.1021G>A (p.G341R)	heterozygous
681	*PHEX*	chrX:22051133	NM_000444:c.10G>C (p.E4Q)	hemizygous
681	*BBS7*	chr4:122780163	NM_176824:c.512T>G (p.V171G)	heterozygous
681	*BHLHA9*	chr17:1174056	NM_001164405:c.199C>A (p.R67S)	heterozygous

681	*CD244*	chr1:160811575	NM_001166663:c.178C>T (p.Q60X)	heterozygous
681	*CILP*	chr15:65489706	NM_003613:c.2918T>C (p.M973T)	heterozygous
681	*COMP*	chr19:18899680	NM_000095:c.571T>C (p.C191R)	heterozygous
681	*DCHS1*	chr11:6651439	NM_003737:c.4586G>T (p.R1529L)	heterozygous
681	*DNAH5*	chr5:13841169	NM_001369:c.5555A>T (p.D1852V)	heterozygous
829	*GPHN*	chr14:67579841	NM_020806:c.1579G>A (p.E527K)	heterozygous
829	*HGD*	chr3:120352019	NM_000187:c.1163C>T (p.P388L)	heterozygous
829	*KIF1C*	chr17:4926781	NM_006612:c.2647delG (p.E883Kfs*204)	heterozygous
829	*LBR*	chr1:225591161	NM_002296:c.1692T>G (p.F564L)	heterozygous
829	*LZTR1*	chr22:21351254	NM_006767:c.2405A>G (p.K802R)	heterozygous
829	*MAPKBP1*	chr15:42113915	NM_001128608:c.2870C>T (p.P957L)	heterozygous
829	*MTO1*	chr6:74210345	NM_001123226:c.2086A>G (p.N696D)	heterozygous
829	*MYL1*	chr2:211155786	NM_079420:c.569A>G (p.H190R)	heterozygous
829	*PIGG*	chr4:509834	NM_001345986:c.707C>T (p.P236L)	heterozygous
829	*POR*	chr7:75615279	NM_000941:c.1708C>T (p.R570C)	heterozygous
829	*ROR2*	chr9:94495662	NM_004560:c.679G>A (p.A227T)	heterozygous
829	*SPEF2*	chr5:35641685	NM_024867:c.314A>C (p.Q105P)	heterozygous
829	*ACAN*	chr15:89392891	NM_001369268:c.1955C>T (p.Thr652Met)	heterozygous
829	*AGBL1*	chr15:86697756	NM_152336:c.358C>T (p.His120Tyr)	heterozygous
829	*ARHGEF 10*	chr8:1871748	NM_001308153:c.2446G>A (p.Gly816Arg)	heterozygous
829	*CEP112*	chr17:64173070	NM_001353127:c.175G>A (p.Gly59Arg)	heterozygous
829	*COL12A 1*	chr6:75841699	NM_004370:c.5894G>A (p.Arg1965His)	heterozygous

829	*CYP3A4*	chr7:99377637	NM_017460:c.143G>A (p.Gly48Glu)	heterozygous
829	*DHPS*	chr19:12792487	NM_001930:c.94C>T (p.Arg32Trp)	heterozygous
829	*DHX38*	chr16:72130893	NM_014003:c.496C>T (p.Arg166Cys)	heterozygous
829	*GLI3*	chr7:42063116	NM_000168:c.1448A>G (p.His483Arg)	heterozygous
338	*ITPR3*	chr6:33627297	NM_002224:c.682C>T (p.Arg228Trp)	heterozygous
338	*NANS*	chr9:100823121	NM_018946:c.190C>T (p.Arg64Trp)	heterozygous
338	*PTPN23*	chr3:47448830	NM_015466:c.896G>A (p.Arg299His)	heterozygous
338	*RBMX*	chrX:135957688	NM_002139:c.598C>G (p.Pro200Ala)	hemizygous
338	*SCN4A*	chr17:62049987	NM_000334:c.215C>T (p.Pro72Leu)	heterozygous
338	*SLC39A4*	chr8:145638738	NM_017767:c.1435G>A (p.Asp479Asn)	heterozygous
338	*SPTAN1*	chr9:131388886	NM_001375318:c.6532C>T (p.Arg2178Cys)	heterozygous
338	*USP18*	chr22:18653541	NM_017414:c.745C>T (p.Pro249Ser)	heterozygous
338	*FARSB*	chr2:223489442	NM_005687:c.953T>G (p.Val318Gly)	heterozygous
338	*FIG4*	chr6:110146307	NM_014845:c.2563T>C (p.Phe855Leu)	heterozygous
338	*GNAT2*	chr1:110151256	NM_005272:c.458C>T (p.Ser153Phe)	heterozygous
338	*GUCY2C*	chr12:14766127	NM_004963:c.3146G>A (p.Arg1049Gln)	heterozygous
338	*H6PD*	chr1:9305475	NM_004285:c.482G>A (p.Arg161Gln)	heterozygous
338	*IGSF3*	chr1:117156788	NM_001007237:c.431A>G (p.Asp144Gly)	heterozygous
338	*LAMA1*	chr18:6977817	NM_005559:c.6254T>A (p.Leu2085X)	heterozygous
338	*LDHD*	chr16:75148574	NM_153486:c.479C>T (p.Ala160Val)	heterozygous
338	*MACF1*	chr1:39783012	NM_012090:c.3730C>T (p.Arg1244Cys)	heterozygous
338	*NEB*	chr2:152432756	NM_001271208:c.16817A>G (p.Tyr5606Cys)	heterozygous

338	*NRIP1*	chr21:16340221	NM_003489:c.293G>A (p.Arg98Gln)	heterozygous
338	*PDGFRA*	chr4:55144678	NM_001347830:c.2191C>T (p.Arg731Trp)	heterozygous
338	*RIC1*	chr9:5763642	NM_020829:c.2615A>C (p.Glu872Ala)	heterozygous
338	*ROR2*	chr9:94495617	NM_004560:c.724C>T (p.Arg242Cys)	heterozygous
