# Supplementary material for: Dose Dependence Effect in Biallelic WNT10A Variant-Associated Tooth Agenesis Phenotype
Source: Diagnostics (Basel). 2022 Dec 7;12(12):3087. doi: 10.3390/diagnostics12123087 (PMC9776737; doi:10.3390/diagnostics12123087)
Supplement: Supplementary file 1 [file diagnostics-12-03087-s001.zip › Table S2.pdf]

Table S2. The primer sequences used for PCR in this study.

Primers	Sequences (5’-3’)	Products (bp)
Exon 1-Forward	AGTAGGCGCCTAACAGCTAG	259
Exon 1-Reverse	TCTGATTTCCTTCCTGCCCA	259
Exon 2-Forward	TCCTCTAACTGGGTCTTTGC	414
Exon 2-Reverse	CAGAGGCGGAGTGTCTAAGT	414
Exon 3-Forward	AGCTCTTCCACGGCTTCT	379
Exon 3-Reverse	TCCACATTCTCTCCTGGTCT	379
Exon 4-1-Forward	GTGTGTGTCGGGGGAAGG	450
Exon 4-1-Reverse	CAGGTTGTTCAAGTTATTCAGG	450
Exon 4-2-Forward	CCTGTCATCACAGAGCATGT	445
Exon 4-2-Reverse	CTTTCTTTAGTGCCCACGAC	445
